# Books and Magazines

**Published:** 1898-04

**Authors:** 


Bulletin of Medical Publications issued by W. B. Saunders, No. 925 Walnut Street, Philadelphia.

Just ready. A new volume in the "American Text-Book" series. An American text-book of Genito-Urinary and Skin Diseases (including syphilis). Edited by L. Bolton Bangs, M. D., late Professor of Genito-Urinary and Venereal Diseases in the New York Post-Graduate Medical School and Hospital; and Wm. A. Hardaway, M. D., Professor of Diseases of the Skin in the Missouri Medical College. Complete in one handsome imperial octavo volume of over 1000 pages, beautifully illustrated. Prices: Cloth, $7 net; sheep or half morocco, $8 net.

**In Preparation for Early Publication.**

An American Text-Book of Diseases of the Eye, Ear, Nose, and Throat. Edited by G. E. de Schweinitz, M. D., Professor of Ophthalmology in the Jefferson Medical College,

Philadelphia; and B. Alexander Randall, M. D., Professor of Diseases of the Ear in the University of Pennsylvania and in the Philadelphia Polyclinic.

AN AMERICAN TEXT-BOOK OF PATHOLOGY. Edited by John Guiteras, M. D., Professor of General Pathology and of Morbid Anatomy in the University of Pennsylvania; and David Riesman, M. D., Demonstrator of Pathological Histology in the University of Pennsylvania.

AN AMERICAN TEXT-BOOK OF LEGAL MEDICINE AND TOXICOLOGY. Edited by Frederick Peterson, M. D., Clinical Professor of Mental Diseases in the Woman's Medical College, New York; Chief of Clinic, Nervous Department, College of Physicians and Surgeons, New York; and Walter S. Haines, M. D., Professor of Chemistry, Pharmacy, and Toxicology in Rush Medical College, Chicago, Illinois.

STENGEL'S PATHOLOGY, a manual of Pathology. By Alfred Stengel, M. D., Instructor in Clinical Medicine, University of Pennsylvania; Physician to the Philadelphia Hospital; Professor of Clinical Medicine, Woman's Medical College; Physician to the Children's Hospital; late Pathologist to the German Hospital, Philadelphia, etc.

CHURCH AND PETERSON'S NERVOUS AND MENTAL DISEASES. By Archibald Church, M. D., Professor of Mental Diseases and Medical Jurisprudence in the Northwestern University Medical School, Chicago; and Frederick Peterson, M. D., Clinical Professor of Mental Diseases in the Woman's Medical College, New York; Chief of Clinic, Nervous Department, College of Physicians and Surgeons, New York.

HEISLER'S EMBRYOLOGY. A Text-Book of Embryology. By John C. Heisler, M. D., Professor of Anatomy in the Medico-Chirurgical College, Philadelphia.

KYLE ON THE NOSE AND THROAT. Diseases of the Nose and Throat. By D. Braden Kyle, M. D., Chief Laryngologist to St. Agnes' Hospital; Bacteriologist to the Orthopedic Hospital and Infirmary for Nervous Diseases; Instructor in Clinical Microscopy and Assistant Demonstrator of Pathology, Jefferson Medical College, Philadelphia.

HIRST'S OBSTETRICS. A Text-Book of Obstetrics. By Barton Cooke Hirst, M. D., Professor of Obstetrics in the University of Pennsylvania.

WEST'S NURSING. An American Text-Book of Nursing. By American Teachers. Edited by Roberta M. West, late Superintendent of Nurses in the Hospital of the University of Pennsylvania.

THE LIVING AGE needs but to be read to be appreciated. Elevating, entertaining and instructive, it embraces every department of literature, including some of the best fiction of the day and poetry, and contains something for every variety of taste.

The following partial contents of its February issues is suggestive of its wide scope and great value. It is indeed invaluable to one who has neither time nor opportunity for scanning all the magazines but who is desirous of keeping abreast of the literary current.

Most of its articles are of great present interest, as well as of permanent value, yet they can be obtained in no other way except by subscribing direct for the periodicals in which they originally appear, and these are many and various, comprising not only those of Great Britain but many of France, Germany, Spain and other continental sources. For instance—"The Degredation of Dreyfus," from the French of Adolph Brisson, in *Les Annales;* "A Session of the Reichstag," from the German of Richard Nordhausen, in *Ueber Land und Meer;* "The Coming of the Slav," by Geo. Washburn, D. D., in *Contemporary Review*; "Lewis Carroll," from the *Spectator;* "The Higher Education of Women in Russia," by Princess Kropotkin; "A Walk thro' Deserted London," by Sir Algernon West; "A Simple Story," from the Polish of M'me Marguerite Poradowzka; "A Lady's Life on a Ranch," by Moira O'Neill; "Pilgrims and Emigrants," from the French of Emile Bertaux; "A Woman Learned and Wise," by Alexander H. Japp; "Burns," by Charles Whibley—and many others, with fiction, including an installment in each number of "With all Her Heart," a delightful serial, translated for the *Living Age* from the French of Rene Bazin, and several short stories, and poetry.

The *Living Age* is published weekly, at $6 a year, by the Living Age Co., Boston. Send 15 cents for a sample copy and special offer to new subscribers.

---

THE principal contributed article in the *American Monthly Review of Reviews* for April is entitled "Political Germany," and was written expressly for the *American Monthly* by Dr. Thedor Barth, the eminent German publicist, leader of the Liberals in the Reichstag, and editor of the *Nation*. Dr. Barth is well known in the United States, having frequently visited here. His article is illustrated with the portraits of all the representative leaders in modern German politics, and is altogether the most complete and lucid exposition of the latest problems and policies of German statesmanship that has yet appeared. Baron Pierre de Coubertin, the brilliant young French writer, contributes to the same number a suggestive article in answer to the question, "Does Cosmopolitan Life Lead to International Friendliness?" Some interesting views of Cannes, where Mr. Gladstone spent the first half of the winter, are published in connection with the article. Dr. Albert Shaw also appends a sketch of M. de Coubertin's own life and achievements in promoting the highest type of internationalism.

